# An Uncommon Cause of Acute Heart Failure: A Case Report

**DOI:** 10.7759/cureus.69509

**Published:** 2024-09-16

**Authors:** Fábio Pé D’Arca Barbosa, Mariana Martinho, Erica Barata, Vanda Spencer, Paula Fazendas

**Affiliations:** 1 Department of Internal Medicine, Unidade Local de Saúde de Almada-Seixal, Almada, PRT; 2 Department of Cardiology, Unidade Local de Saúde de Almada-Seixal, Almada, PRT

**Keywords:** brain infarction, heart failure, hypereosinophilic syndrome, venous thromboembolism, ventricular dysfunction

## Abstract

Hypereosinophilic syndromes (HES) comprise a group of rare disorders characterized by persistent blood eosinophilia, accompanied by eosinophil-associated organ damage. Nearly every organ can be affected, with the skin, lungs, heart, and gastrointestinal tract being the most common. Unspecific presenting symptoms hinder timely management, facilitating disease progression. Treatment is aimed at the underlying disease, reducing disease progression and controlling symptoms. We present the case of a 39-year-old female admitted with an acute venous thromboembolic event who developed acute hypereosinophilic-related heart failure and encephalopathy. After a thorough investigation, the diagnosis of idiopathic HES with multiorgan involvement was established, and the patient was medicated with high-dose corticosteroids, resulting in a good clinical and laboratory response. Due to non-compliance with medical treatment, disease progression culminated in death. This case highlights the multiorgan involvement in HES and the importance of treatment adherence for these patients.

## Introduction

Hypereosinophilia is defined based on an absolute eosinophil count (AEC) greater than 1500/mm³ on two consecutive occasions and persistent for a minimum of one month (although this time gap can be shortened in severe cases) [1].

In hypereosinophilic syndromes (HES), sustained eosinophil production causes damage to multiple organs through eosinophilic infiltration and mediator release. Activated eosinophils have pro-inflammatory effects, either due to direct cytotoxicity to tissues or by endothelial cell activation, promoting thrombosis, fibrosis, angiogenesis, and tissue remodelling [2].

AEC does not correlate linearly with the severity of HES, as tissue infiltration and organ damage by eosinophils may perdure despite lowering the blood eosinophilia; therefore, adequate follow-up should not solely rely on AEC vigilance [2].

Aetiological investigation should rule out myeloid and lymphoid neoplasms, as eosinophil growth and accumulation can either be due to an intrinsic defect of eosinophil-committed neoplastic progenitor cells, caused by mutations including platelet-derived growth factor receptor (PDGFR) or fibroblast growth factor receptor 1 (FGFR1), or cytokine overproduction, such as interleukin-3 (IL-3) and IL-5, that stimulate the growth, differentiation, and survival of eosinophils. Secondary causes, such as fungal and parasitic infections, should also be taken into consideration [2].

Nearly every organ can be affected by HES. Neurologic and cardiac involvements (Loeffler’s endocarditis) are major causes of morbidity and mortality in patients with HES. Myocardial damage occurs with varying levels of severity and can lead to fibrosis [3].

Treatment aims to reduce eosinophil count, control symptoms, and prevent disease progression. This is usually achieved with either corticosteroids, cytoreductive agents, or antineoplastic agents, depending on the underlying condition. In patients with Loeffler’s endocarditis, early steroid treatment inhibits progression to the fibrotic stage and improves cardiac function [2]. In the fibrotic state, hypereosinophilic myocarditis usually has a poor prognosis.

## Case presentation

**Figure 1:**
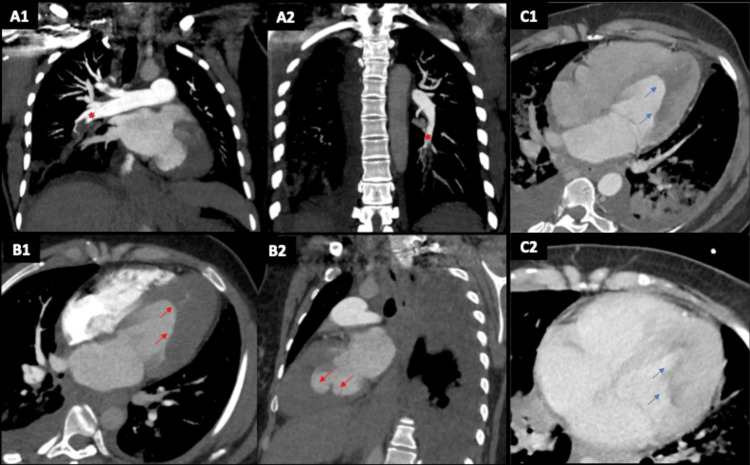
Computed tomography with pulmonary angiography These images reveal multiple bilateral lobar pulmonary embolisms (panels A1-A2, red asterisks) and also obliteration of the left ventricular apex (panels B1-B2, red arrows) by a subendocardial hypodensity structure, highly suspicious of being a thrombus, after early arterial and delayed contrast CT phases (panels C1-C2, blue arrows).

A 36-year-old female presented to the Emergency Department with a right submandibular painful mass and a swollen right leg. Initial investigation revealed the presence of a peritonsillar abscess and right popliteal venous thrombosis. The patient was started on intravenous amoxicillin and clavulanate, and anticoagulation with subcutaneous enoxaparin 1 mg/kg 12-hourly. Blood work revealed marked eosinophilia (14.43 x 10^9/L) not otherwise explained. A thorough past medical history investigation revealed an unspecified oesophageal pathology, as well as an unspecified pulmonary condition and skin rash four years prior to admission, for which the patient was medicated with corticosteroids, having improved. She had also been exposed to mould for several years due to her job and had lived in both Guinea-Bissau and Brazil in the past. Shortly after hospital admission, the patient developed sudden dyspnoea and hypoxemia. She maintained haemodynamic stability, and blood tests revealed increased high-sensitivity troponin T of 870 ng/L and NT-pro brain natriuretic peptide of 16,600 pg/mL. A bilateral lobar pulmonary embolism was confirmed after a computed tomography pulmonary angiogram (CTPA). As CTPA images also showed myocardial thickening, chest CT with an early arterial contrast phase was performed and revealed a left ventricular (LV) thrombus (Figure 1).

**Figure 2:**
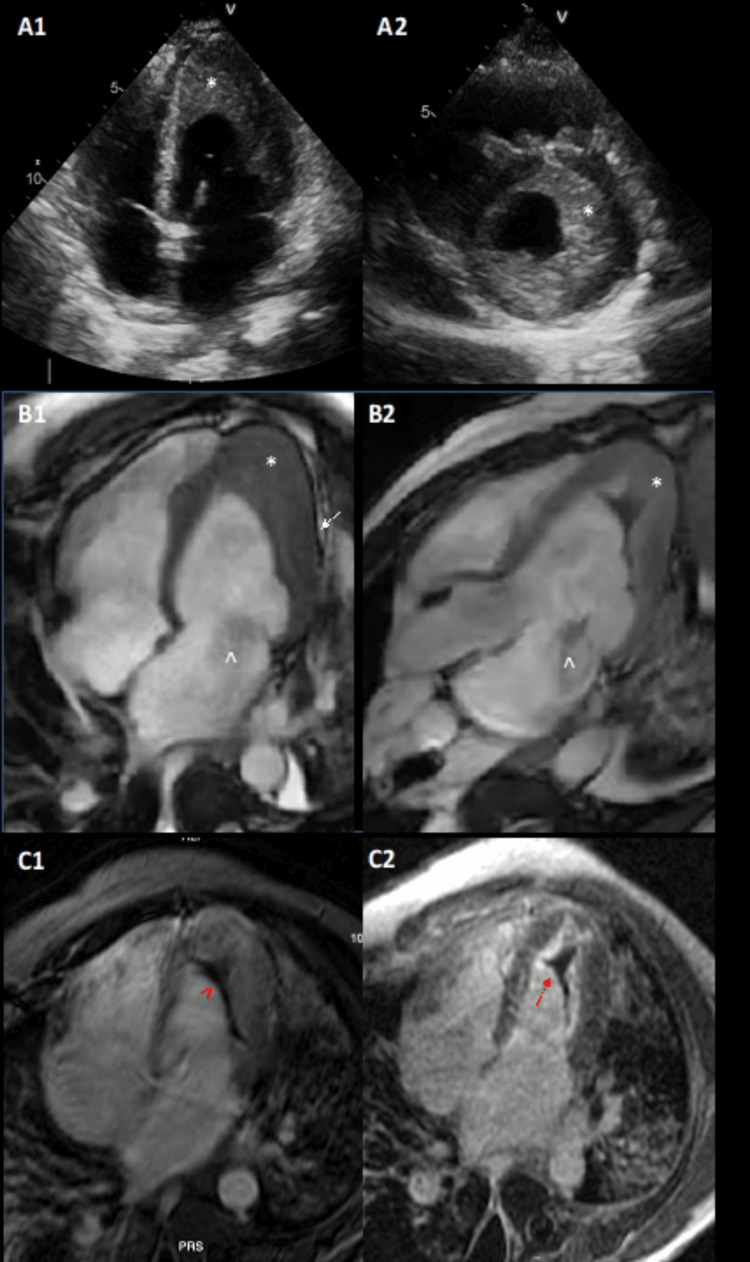
Transthoracic echocardiogram and magnetic resonance These images reveal apical four-chamber and short-axis views (panels A1-A2, respectively), showing a reduced LV cavity due to a hyperechogenic structure apparently contiguous to the myocardium and involving all apical segments, as well as the medial segments of the anterior, anterolateral, inferolateral, and inferior walls (white asterisks). Panels B1-B2 show cine cardiac magnetic resonance images, with almost total obliteration of the left ventricle apex, non-quantified mitral regurgitation (white arrowhead), and mild pericardial effusion (white arrow). Left ventricular thrombus was confirmed by early gadolinium enhancement (panel C1, red arrowhead), and late gadolinium enhancement confirmed endomyocardial fibrosis with the pathognomonic 3 layers V sign, composed of thrombus, endomyocardial fibrosis, and myocardium (panel C2, red arrow).

The patient underwent a full cardiac investigation with transthoracic echocardiogram and magnetic resonance imaging (MRI), which were compatible with Loeffler endocarditis, including bi-ventricular systolic dysfunction, LV thickening, severe endomyocardial fibrosis, and apical LV thrombus (Figure 2).

Left heart failure symptoms ensued, and the patient was started on inotropic support, diuretic therapy, and non-invasive ventilatory support, with clinical stabilization and improvement.

Due to findings of non-specific oesophageal thickening on the chest CT scan, the patient was submitted to upper digestive endoscopy, and biopsies were performed. Histological evaluation was non-conclusive.

**Figure 3:**
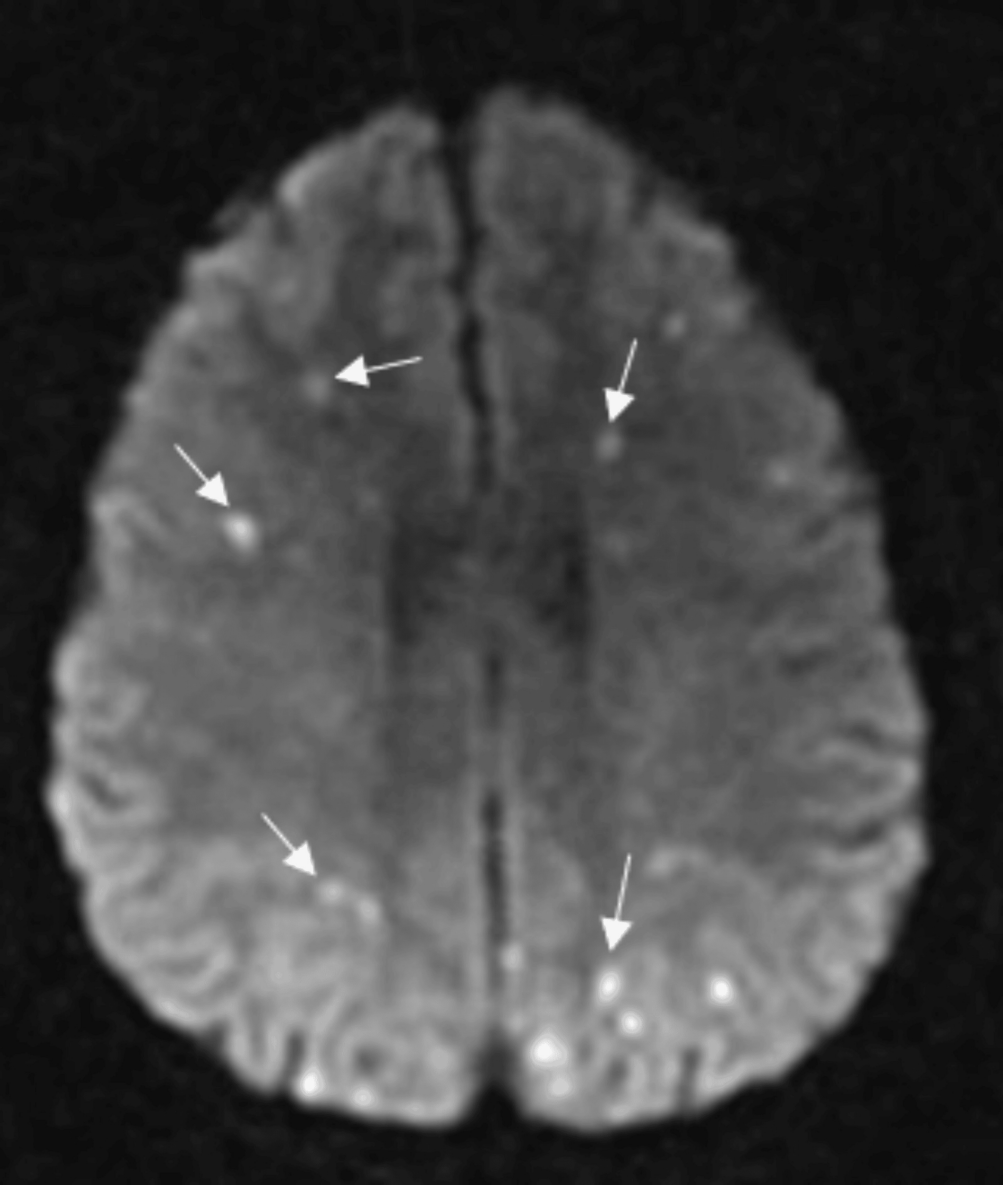
Diffusion-weighted magnetic resonance imaging of the brain This image shows evidence of acute vascular events, such as focal hyperintensities (white arrows). The bilateral distribution and involvement of multiple vascular regions suggest an embolic source.

During hospital admission, the patient also developed an encephalopathic state and had minor focal neurological deficits, which is why she was submitted to brain CT scans and MRI studies, revealing findings consistent with multiple, bilateral, ischaemic strokes (Figure 3). The patient was then started on a rehabilitation programme.

For the aetiological study of hypereosinophilia, a genetic study for the detection of FIP1-Like 1 (FIP1L1)-PDGFRA, PDGFRB, FGFR1, Janus Kinase 2 (JAK2) Valine 617 Phenylalanine (mutation) (V617F), and Breakpoint Cluster Region/Abelson (BCR/ABL) was negative. Bone marrow biopsy and myelogram showed a predominance of eosinophils (33%) without myelodysplasia or blast cells. Bronchoalveolar lavage mycologic studies were negative. Stool specimens’ analysis was negative for parasitic infections. Blood cultures and serologies for fungal and helminthic infections, such as *Strongyloides*, *Toxocara*, and *Trichinella*, were negative. Total IgE levels were elevated (577 U/mL).

Considering the diagnosis of HES with multiorgan involvement, namely cardiac, vascular, neurological, and possibly gastrointestinal, the patient was started on corticosteroids (methylprednisolone 1 g, two pulses, followed by oral prednisolone 1 mg/kg/day), with very good laboratory response (achieving AEC of 0.08 x 10^9, 0.4%) and clinical improvement, resolving the encephalopathic state and allowing for weaning of inotropic and respiratory support. The patient was discharged home under corticotherapy with prednisolone 60 mg/day and anticoagulation with rivaroxaban 20 mg/day. However, due to therapeutic non-compliance and disease progression, she was readmitted a few days later and died of decompensated heart failure.

## Discussion

HES can affect nearly every organ and presents with non-specific symptoms, which delays diagnosis and allows for disease progression, as was the case with this patient, who had symptoms for at least five years prior to diagnosis. This case study demonstrates the wide range of organ involvement in HES, as shown by cardiac, vascular, neurological, and possibly digestive tract involvement during hospital admission, and possible skin and pulmonary involvement in the past.

Cardiac involvement, either by infiltration of eosinophils in the myocardial wall or by the presence of an intra-cardiac thrombus, is a major cause of mortality in this population [3]. The patient presented in this article had a good clinical response to pharmacological therapy directed at both heart failure and HES.

Vascular involvement in HES can affect both venous and arterial systems [1,2]. Despite being anticoagulated since admission, our patient still developed embolic events. This can be due both to the inefficiency of anticoagulation in preventing new events in these patients and to other mechanisms being involved. Research is ongoing to fully describe the pro-thrombotic mechanisms in HES.

Neurological involvement in HES can manifest as encephalopathy, embolic strokes, and peripheral neuropathy [1,2]. In this case study, the encephalopathic state resolved with corticotherapy, and imaging studies confirmed the presence of ischemic strokes.

Gastrointestinal involvement could not be confirmed in this patient, probably due to inadequate specimen collection, as biopsy specimens were too superficial and the results were not conclusive. Skin and pulmonary involvement could have occurred in the past, having resolved with corticotherapy.

The aetiological investigation is crucial, as treatment is directed towards the cause of eosinophilia. In cases where an infection is present, starting corticosteroids before anti-helmintic, protozoal, antibacterial, or fungal agents can be detrimental.

Non-adherence to therapy allowed for disease progression and culminated in refractory cardiac and respiratory failure, highlighting the impact of treatment on symptom control and mortality suppression.

## Conclusions

This case illustrates the complexity and severity of HES, particularly when involving multiple organ systems. Early and accurate diagnosis is crucial, as delayed treatment can lead to irreversible damage. This patient had significant multiorgan involvement, with cardiac, vascular, and neurological manifestations. Initial treatment with corticosteroids and supportive care showed a positive response, emphasizing the importance of timely intervention in controlling symptoms and preventing further organ damage. However, non-compliance and the advanced stage of the disease ultimately led to a fatal outcome. This case highlights how vast the multiple organ involvement can be in HES and underscores the importance of therapeutic adherence in improving prognosis and preventing mortality.
